# Rheumatism

**Published:** 1899-03

**Authors:** A. L. Kennedy

**Affiliations:** Boston, Mass.


THE
### HOMŒOPATHIC PHYSICIAN,
A MONTHLY JOURNAL OF
HOMŒOPATHIC MATERIA MEDICA AND CLINICAL MEDICINE.

If our school ever gives up the strict inductive method of Hahnemann, we are lost, and deserve only to be mentioned as a caricature in the history of medicine."—CONSTANTINE HERING.

Vol. XIX. MARCH, 1899. No. 3.

### PROCEEDINGS OF THE SOCIETY OF HOMŒOPATHICIANS,

### BUREAU OF CLINICAL MEDICINE.

F. W. PATCH, *Chairman.*

### RHEUMATISM.

BY A. L. KENNEDY, M. D., BOSTON, MASS.

Rheumatism—what mingled feelings of horror and pity stir the heart at the mention of this name! The thought pervades all classes, the high and the low, the rich and the poor, the dwellers in the mountains and the inhabitants of the plains. It is well-nigh universal, for all are alike subject to the disease, and few may escape its baleful attacks.

Unlike many another dread enemy of man's comfort that seemingly lies in wait like a cowardly foe in ambush watching an opportunity to spring upon the unsuspecting, or, waiting to attack the sufferer already beset by others, this bold disturber of human rest, disdaining help from allies, fearlessly chooses its victims from among the robust, and even evinces a preference for those in early manhood and womanhood, in whom the life current runs full and strong.

The knowledge that one is afflicted with rheumatism seems to carry with it the conviction—a sort of foregone conclusion—that that one is suffering from an incurable disease, and this opinion is held regardless of the nature or type in question.

That the people should hold such views respecting this affliction is not strange when one considers the attitude of the medical fraternity in the aggregate toward the same condition.

We are all familiar with the story told of the sufferer from chronic rheumatism who consulted a prominent physician of wide experience, with a view to receiving treatment. The would-be patient was most graciously received, but upon making known the nature of his ailment had his attention called very unceremoniously to the space over the office door where rested the doctor's trusty rifle, and at the same moment the voice of the sagacious follower of Æsculapius rang in his waiting ear: "Do you see that gun? Get out of my office as quick as the Lord will let you; if you don't, I'll shoot you."

This incident, though perhaps legendary, yet serves to illustrate the attitude of the dominant school of medicine of to-day toward this troublesome and intractable malady.

With the medical profession holding such views it is not to be wondered at that the laity should be considerably exercised at the idea of a possible attack, for it is a notable fact that the people are not slow to interpret and voice the feelings of the profession toward a prevailing disorder, nor is it to be thought strange that a school of medicine holding the opinion that the cause of disease must of necessity be a *materies morbi* should realize its inability to cope with the malady, the real ætiology of which is entirely unknown. Once given a thorough acquaintance with the determining *cause* of this disorder and the method of procedure is comparatively plain (?). Is it an excess of alkali in the blood?—correct it by administering a due amount of acid. Is there already too much acid in the system?—then it should be met and overcome by a sufficiently vigorous alkaline treatment. And if it could only be discovered by some enterprising aspirant for notoriety in the medical world that all infirmities of this nature were due to some wandering member of the microbe family who, separated from his fellows and unable to rejoin the "flying squadron," is yet determined to let his energies be felt by improving every opportunity to run the blockade and effect a landing on susceptible soil. If such could be the case what a boon to humanity! But alas! an afflicted world still waits for the microbe; but be not disheartened, good people, for even now there are rumors that the trail of the serpent has been discovered, and who knows but that ere long this subtle enemy, this wary strategist will be found, only to be speedily "bottled up" for due inspection and disposition becoming his rank.

What a travesty on medicine! We will not trespass, gentlemen, upon your patience, nor use your valuable time on this occasion, to recount the numerous theories of rheumatism that have successively if unsuccessfully flourished from time to time since the earliest ages. Their name is legion; their history is spread upon the pages of our medical books; whosoever will may read—suffice it to mention at this time two or three only. One, the germ theory, which to-day is gaining so many adherents on other lines, seems to have made but little progress here. In regard to this the *American Year-Book of Medicine and Surgery* for 1898, referring to examinations made for bacilli with the view of determining the infectiousness of the disease, says: "Cultures varied in results, though those made from the fluid in the joint and also from neighboring glands both showed a bacillus. This was regarded as evidence of the infectiousness of the disease. Yet as many before and since have discovered bacilli no reliance can be placed on any of the claims to the discovery of the specific bacillus." Of this theory Osler says: "Investigations thus far have shown no constancy of any micro-organism in the disease." Another, the nervous theory, is said to have many advocates who regard as analogous, locomotor ataxia, chorea, and the arthropathics of myelitis; but the theory that perhaps of late years has the most adherents is the so-called metabolic, implying the presence in the system of morbid material, the result of defective assimilation, and this material, it has been suggested, is Lactic-acid or Lactic-acid with certain combinations. This latter theory was first advanced a little more than two decades ago, and while still advocated to some considerable extent yet seems doomed like its predecessors to be eventually discarded.

And in the matter of treatment the old school is even less united (if possible) than in the direction of ætiology, but lest I be suspected of prejudice on the point, I transcribe, for the benefit of any who might be so exercised, the words of Prof. Austin Flint, on pp. 1096 and 1097 of the fifth edition of his treatise on *Principles and Practice of Medicine*, published in 1884. He says: "There are few diseases in nosology, the treatment of which, during the last half century, has undergone such mutations as that of acute articular rheumatism. Among the measures more or less in vogue during the period just named are the following: Bleeding, general and local, Mercurialization, Colchicum carried to the extent of producing vomiting and purging, Nitrate of Potassa, an ounce or more given daily, Opium in large doses, large doses of Quinia, and the use of Veratrum-viride. Cases in which reliance has been chiefly placed on each of these measures have pursued a favorable course, and the treatment has seemed to be successful. Want of knowledge of the natural history of the disease—that is, where it has been allowed to pursue its course under favorable hygienic circumstances, uninfluenced by therapeutical interference—has heretofore rendered it difficult to judge of the effect of different methods of treatment. My report of thirteen cases, and the report by Dr. Sutton of a larger number, treated with only palliative remedies, show an intrinsic tendency in the disease to end within a shorter period, and a less degree of liability to complications than have been hitherto generally supposed. Hence it may be reasonably concluded that the influence of different methods of treatment over the duration of the disease and the occurrence of complications has been over-estimated. Each of the measures above enumerated probably is, to a certain extent, useful in certain cases, but a special controlling influence over the disease is exerted by none, and clinical observation has shown the development of endocarditis, pericarditis, and other complications under treatment by each of them. It is not worth while, therefore, to discuss their merits and demerits severally, inasmuch as few, if any, practitioners now rely upon any one of them." And Osler, in his work recently published, regards the matter in much the same light. He says: "Medicines have little or no control over the duration or course of the disease." And referring to the recent advocacy of Salicylic-acid and its compounds he further says: "Salicyl compounds act chiefly to relieve pain." Other works consulted, including Reynolds, Pepper, Strümpell, Keating, *Reference Handbook of Medical Sciences*, *American Text-book of Applied Therapeutics* for 1896, *American Year-Book of Medicine and Surgery* for 1898, and Gould T. Vint, confirm these views. To the mind honestly striving for something reliable upon which to base a system of treatment, but bewildered in its search by the multitude of conflicting and ever-varying theories that greet him on every page, how refreshing to turn to Hahnemann and note with what assurance he defines the course to be pursued in order to furnish the best results! While his colleagues are striving to determine the particular form of the bacillus for each phase of the disease, respectively, that they may know what drugs to employ, Hahnemann recognizes no material thing as the essential cause, but with the knowledge that there is a disturbance of the vital force proceeds to apply to cases of all description, to all manner of ills to which mankind is heir, including that under consideration, the one principle of therapeutics, the one law of cure, with the satisfaction of showing by far the best results of any method of practice thus far employed, and the experience of his followers with their tens of thousands of patients for nearly a century has served to confirm the superior excellence of his system.

In a personal experience of over twenty years I have found the same natural law to be applicable in the therapeutic management of *this* as in all other human ailments that have thus far fallen under my observation. Not all the cases of rheumatism that I have treated have been restored to perfect health, but my success has been such as to warrant me in regarding the results as most gratifying. Cases of an acute nature have yielded promptly to the indicated remedy. We are taught that one attack of rheumatism predisposes to another. But in our experience with that phase known as rheumatic fever we fail to recall an instance where there has been a subsequent attack, and this notwithstanding, in some cases, severe exposure. Some of these patients had previously suffered from repeated attacks.

Treatment of cases of a sub-acute and chronic nature—as might be expected—has not shown as satisfactory results. This, I believe, may be attributed to various reasons. The nature of the disease (possibly in some case incurable), the lack of patience on the part of the patient, and the inability on the part of the prescriber to select the appropriate remedy.

Some of these, however, have responded beautifully, which fact is certainly a very suggestive one, and one fraught with hope for many a poor sufferer.

Remedies: Of remedies we have used several as called for, and each in turn has served us well. We would call your attention to but one, and of that we can scarcely speak too highly. It has helped me greatly in controlling cases characterized by migratory pains. These cases are sometimes very trying, baffling the best efforts of the physician, and keeping him constantly anxious, searching for the appropriate remedy. It is a class of cases in which one is led to consider such remedies as Kali-sulphuricum, Kalmia, Lach., Ledum, Lyco., Puls., Sulphur, et al. The remedy is Lac-caninum. We do not find it referred to in any work on the homœopathic therapeutics of rheumatism, save Lilienthal's. The latter author mentions it briefly, calling attention to its characteristic feature—alternation of sides,— and this is the symptom I have repeatedly verified. Not only is this tendency in the case checked but the whole condition of the patient is speedily and decidedly improved. It would appear that if ever a remedy has a so-called "key-note" then alternation of sides is the key-note of Lac-caninum. This applies not only in rheumatism but in other conditions. In regard to diet I would say that my rule is a light, nourishing, but non-stimulating food. I use some form of cereal as best borne by the individual patient. Fruit and vegetables are desirable, qualified, of course, to suit individual cases. I advise rheumatic patients to refrain from meat, as it has seemed to me they do better without it. In some cases it has seemed best to proscribe even the use of milk. Osler says, "Meat should be used most sparingly." To sum up, in conclusion, we note, First, of rheumatism, considered ætiologically we to-day know nothing. Second, rheumatism in its acute form is curable. Third, rheumatism once cured is not likely to recur.

### DISCUSSION.

Dr. Kimball—I think any one of us would rather attend to a case of rheumatic fever than a case of chronic rheumatism. Acute cases will get well, usually, chronic ones are doubtful, to say the least. In regard to the diet I think it is much more important in the acute cases than in the chronic. I always restrict meat entirely, also the use of milk, on account of the Lactic-acid it contains, and acids generally. I have seen decided aggravations occur on account of taking a lamb chop or a glass of milk. In acute cases where fever is not high, and there are no indications of disturbances of digestion, potatoes, fish, cocoa, bread and butter, etc., can be given. If there is much fever the diet is rather unpleasant for the patient, being chiefly, for my patients, water gruels. As I do not want them to eat at all until they are hungry, they are usually hungry enough to take it. In chronic cases I do not believe the diet has as much effect. I forbid the excessive use of meats such as mutton and beef, also acids, and of course stimulants should be avoided in all cases.

Dr. Carleton—That was an orthodox paper from a homœopathic standpoint. I have been interested in the use of Lactic-acid, which is especially indicated if there is a bright, shining, glistening throat. Many cases cannot take milk; some can. Some who cannot take it plain take it scalded. Some who cannot take it in that form can take koumiss. In chronic cases, what agrees with one is poison to another. Do not eat or drink that which hurts, but otherwise eat that which agrees. I am glad to see the Doctor keep so near the rules of Hahnemann. If we follow his rules we shall be all right.

Dr. Butler—I rise principally to ask if any have had anything to do with Lactic-acid. It was prophesied that this would have much to do with rheumatism. Has anybody had any favorable or unfavorable experience with it?

Dr. Patch—I have had one case that was cured by Lac-caninum. It was a case that extended over a good many years. At first it seemed to be a Rhus-tox. condition but later on more careful study, Lac-caninum was prescribed. That was some four years ago and there has been no return of the trouble. I believe it to be a complete cure. The patient had had much trouble with the heart, and this also is improved. He is a furniture mover and does hard work. Another case, I do not remember the early history, was taken some four years ago with intermittent fever, which was removed by Belladonna. He was a typical Belladonna subject. The following spring he had no intermittent fever, but a rheumatic fever at the corresponding season. I have never been able to formulate any satisfactory rules of diet for rheumatism. I belong to a rheumatic family myself, and for the last five or six years I have been a strict vegetarian, yet this method of living is no specific against the disease. With patients I have not seen any particular results follow the taking away of meat, although I have been in the habit of doing it. Rheumatism, however, has always seemed to me to be associated with digestive troubles.

Dr. Close—The relation between rheumatism, Lactic-acid, and Lac-caninum is often illustrated. The chemists find Lactic-acid in the blood and in the secretions in rheumatism, and many cases are aggravated by the use of milk. It seems as though some principle which is specific in milk bears a very close relation to rheumatism. Lac-caninum corresponds to a type of cases not infrequently met, in which the pains and inflammation shift from side to side.

Dr. Pease—I can also verify the good work done by Lac-caninum in cases of rheumatism, and by Lactic-acid in gout. The only satisfactory experience I ever had with Lactic-acid was in two cases of gout. The wife of our old friend Dr. Ballard had been troubled with rheumatism in the joints, but there was a peculiar Pulsatilla condition. Pulsatilla was studied and given, and did no more than possibly palliate it. The condition that led to these two remedies was the migratory action of the symptoms, but when I noticed the changing from side to side I gave Lac-caninum with very pleasant results.

Dr. Davis—I have had one case in particular where Laccaninum was curative, and I think one other observer has spoken to-day of a case where he gave Lac-caninum when at first it appeared to be a Rhus-tox. case. My case also appeared at first to be a Rhus case, but Lac-caninum cured. The symptoms were the same as mentioned in the paper. Pain occurred alternately from side to side.

Dr. Pease—I have just been asked what symptoms I noticed in the two cases of gout. Principally the souring of the milk as soon as it reached the stomach. Strangely enough there was a craving for milk in these two cases. That led to the selection of Lactic-acid.

Dr. Kennedy—The diet, I think, is as important in chronic as in acute rheumatism, but in a little different way. Dr. Carleton has suggested that where milk cannot be borne in its raw state, it is borne sometimes when scalded, and salted. I would like to ask Dr. Pease if this was done in those two cases which were so irritated by milk?

Dr. Pease—Yes, sir; in the case where milk was taken.

Dr. Kennedy—Dr. Carleton also said that acute cases would get well provided we would let them alone. I will not dispute that, but will say that if we treat them homœopathically they will get well sooner and the disease is not so likely to recur. I have had no experience with Lactic-acid. If milk is so objectionable, and causes such aggravation in some cases, it would certainly seem as though there was something about it that affected these cases seriously, and the inference is that it is Lactic-acid. Therefore Lactic-acid, given homœopathically, might cure. When a patient suffering from chronic rheumatism is treated homœopathically and relieved, even if he is not cured, he is likely to remain in a very much better state of health ever after. I am glad to hear so many verifications of Lac-caninum. The first time I used Lac-caninum was in one of those cases of alternation of sides. I studied the case as well as I could. The man was a Pulsatilla patient, and he received Pulsatilla, but it did very little for him. He would get slight relief and go back as before. I tried other remedies as closely allied to that as I could find. I consulted Dr. W. P. Wesselhœft and he went over it carefully and said to me: "He has had Pulsatilla?" I said "Yes." He said: "Then give him Lac-caninum." He gave him one little powder. I went to see my patient the next day and found the same old story. I fixed the "second best remedy in the materia medica," and gave him a powder of Lac-caninum on his tongue. The next day I visited him and asked him how he was. He said about the same. He had previously been worse in stormy weather, and the night had been rainy. I asked him what kind of a night he had, and he said fairly good. I fixed some of the "second best remedy" to take regularly. The second day, as I went in, he sat up in bed, took hold of both my hands, and said: "I have not been able to do that for three weeks, have I?" He said that he felt a great deal better. He has never had a return of the trouble.

